# Metagenomic insights into strategies of aerobic and anaerobic carbon and nitrogen transformation in boreal lakes

**DOI:** 10.1038/srep12102

**Published:** 2015-07-10

**Authors:** Sari Peura, Lucas Sinclair, Stefan Bertilsson, Alexander Eiler

**Affiliations:** 1 Department of Ecology and Genetics, Limnology and Science for Life Laboratory, Uppsala University, Uppsala, Sweden; 2 Department of Biological and Environmental Science, University of Jyväskylä, Jyväskylä, Finland

## Abstract

Thousands of net-heterotrophic and strongly stratifying lakes dominate the boreal landscape. Besides their central role as emitters of greenhouse gases, we have only recently begun to understand the microbial systems driving the metabolic processes and elemental cycles in these lakes. Using shotgun metagenomics, we show that the functional potential differs among lake types, with humic lakes being particularly enriched in carbon degradation genes. Most of the metabolic pathways exhibit oxygen- and temperature-dependent stratification over depth, coinciding with shifts in bacterial community composition, implying that stratification is a major factor controlling lake metabolism. In the bottom waters, rare and poorly characterized taxa, such as ε-*Proteobacteria*, but also autotrophs, such as photolithotrophic *Chlorobia* were abundant. These oxygen-depleted layers exhibited high genetic potential for mineralization, but also for fixation of carbon and nitrogen, and genetic markers for both methane production and oxidation were present. Our study provides a first glimpse of the genetic versatility of freshwater anoxic zones, and demonstrates the potential for complete turnover of carbon compounds within the water column.

Humic lakes are important landscape features of the boreal zone. These abundant water bodies are characterized by high concentration of allochthonous dissolved organic carbon and steep gradients in oxygen and nutrients, coupled to strong thermal stratification. Most humic lakes are net heterotrophic and an important source of natural greenhouse gases (GHG) such as CO_2_, CH_4_ and N_2_O^1^,^2^. This type of GHG emissions are coupled to microbial processes in the deep anoxic layers, but there is also biological consumption of GHGs in the water column and, for example in the case of CH_4_ only a minor part reaches the atmosphere^3^. However, ongoing climate change with increasing external carbon inputs and precipitation coupled to prolonged stratification may change these systems profoundly^4^ and this may also result in altered GHG emissions^1^,^5^.

Archaea and Bacteria are the key organisms influencing the balance between the production and consumption of GHG, ultimately controlling such emissions. Since many of these organisms are sensitive to changes in oxygen regime^6^,^7^, changes in lake characteristics and oxygen availability are likely to also alter lake metabolism. Still, before we can predict the ecosystem-scale metabolism of boreal lakes in future scenarios of climate and environmental change, we first need to understand the functioning of contemporary microbial systems.

The results we present here are from a first shotgun metagenomics study analyzing how genetic potential for key metabolic functions, such as methane production and consumption, are distributed between the oxic epilimnion, the oxygen transition zone and the anoxic hypolimnion in three humic lakes. The functional potential in the epilimnion of these humic lakes is further compared to lakes with lower carbon concentration. The humic study systems; Lakes Alinen Mustajärvi, Halsjärvi and Mekkojärvi, are located in southern Finland. They are ice-covered during winter and stratified with regards to oxygen and temperature, except for reoccurring autumn- and less frequent spring-overturns (for lake characteristics, see Table 1).

## Results and Discussion

Samples from the oxic epilimnion, the oxygen transition zone and the anoxic hypolimnion of three humic lakes were characterized by shotgun metagenomics and the representation of marker genes for key metabolic processes were compared. As relatively low amounts of DNA were retrieved from most samples, the DNA extracts were subjected to whole-genome amplification before sequencing. This procedure can potentially skew the distribution of genes or individual genomes compared to the original sample, but the practice of adding DNA to each reaction at concentrations ranging from 10 to 20 ng should minimize the possible representation-bias, which is an accelerating problem with low DNA quantities (<1 ng)^8^. Subsequent 454 pyrosequencing yielded a total of 1.4 Gb of sequence data. After quality filtering 480 Mb of data from nine samples were retained for analysis (Table 1). These data were in 1 176 376 reads, with an average sequence length of 336 bp.

**Figure 1:**
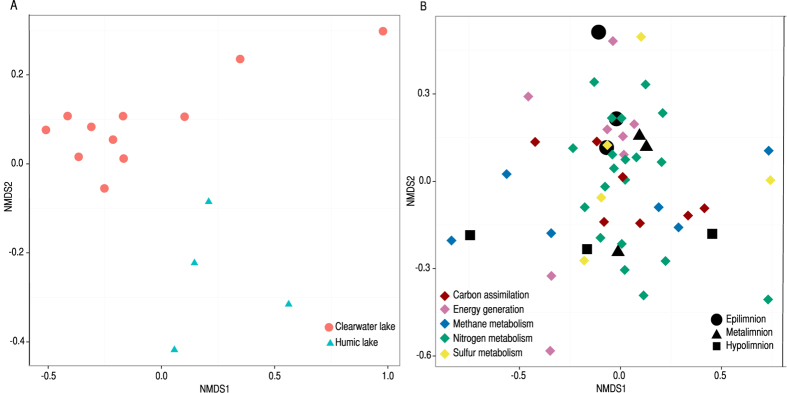
A) NMDS visualizing the difference in functional potential between epilimnia of clearwater and humic lakes. **B**) NMDS visualizing the compartmentalization of key functions between the three layers of Alinen Mustajärvi, Halsjärvi and Mekkojärvi. The plot is showing samples scores (black symbols while shapes represent different layers) and loadings of individual Pfams (diamonds with colors representing different functional blocks).

The nine metagenomes from the three humic lakes and 10 previously analyzed metagenomes from clearwater lakes^9^ were compared based on normalized (genome equivalent) results from blast searches against the STRING database^10^. The comparison showed that previously observed differences in community composition between these two lake types^11^ are also reflected in metabolic potential encoded in the microbial genomes (Fig. 1a, PERMANOVA; pseudo-F = 5.99, p < 0.001). Clusters of orthologous groups^10^ (COGs) from the metagenomes revealed that proteins related to carbon degradation and also groups representing poorly characterized proteins were highly overrepresented in the metagenomes of humic lakes compared to clearwater lakes, reflecting the existing poor understanding of the resident microbiota in these systems (Wilcoxon rank sum test p < 0.05, false discovery rate < 0.056, Table S1). Identified COGs overrepresented in the humic lakes were hydrolases that are related to carbohydrate degradation, such as a beta-xylosidase (COG3664), an enzyme predicted to perform xylanase/chitin deacetylation (COG0726) and an endoglucanase (COG2730). This overrepresentation of COGs involved in cellulose, chitin and starch degradation highlights the fact that the carbon demand in humic lakes relies largely on biopolymer degradation^12^. Also, COGs related to iron uptake were more abundant in humic lake metagenomes. A possible reason for this is the tendency for humic substances to bind free iron, diminishing the bioavailable pool^13^.

Key metabolic pathways in the humic lakes were assessed by using hidden Markov models (HMM) in combination with the Pfam database^14^ (list of marker Pfams in Table S2). These results are presented as occurrences per genome equivalent. Genome equivalent refers to the estimated number of bacterial genomes in each sample, based on the average number of 139 single copy genes^15^ in each individual metagenome. There was a higher similarity in functional potential amongst the three water masses with similar conditions than amongst different layers within individual lakes (Fig. 1b, pseudo-F = 1.71, p < 0.05). The loading plot shows that Pfams related to methane metabolism were one important factor driving the separation between layers.

**Figure 2:**
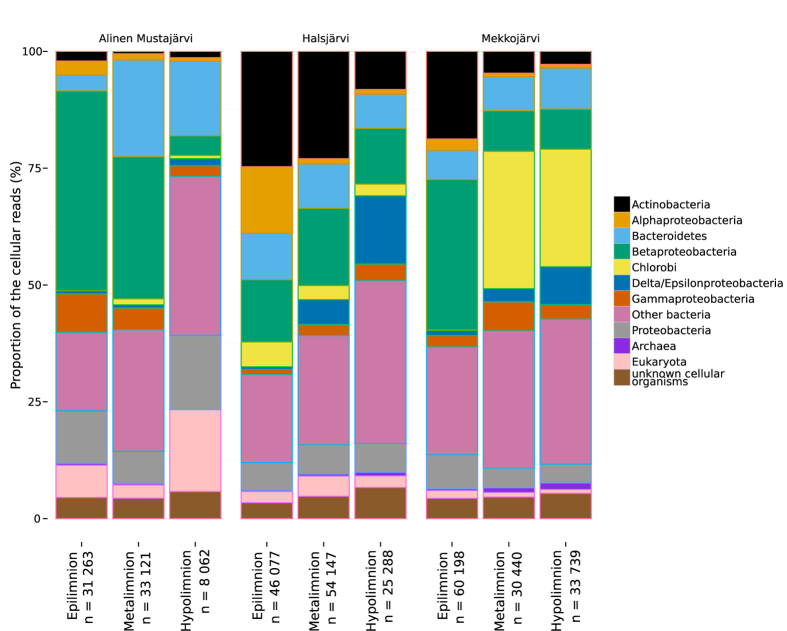
Taxonomic composition of microbiota in the three layers of Alinen Mustajärvi, Halsjärvi and Mekkojärvi derived from all sequences related to cellular organisms. n = number of sequences with hits to cellular organisms in the nr database.

Like the functional profiles, taxonomic composition was distinct among the depth-layers corroborating previous findings from 16S rRNA gene amplicon sequencing^11^. The dominant phyla in the epilimnia were *Actinobacteria* and *Betaproteobacteria*, while the proportion of rare phyla (each contributing less than 3% of the total community) as well as *Chlorobia* increased towards the bottom (Fig. 2).

**Figure 3:**
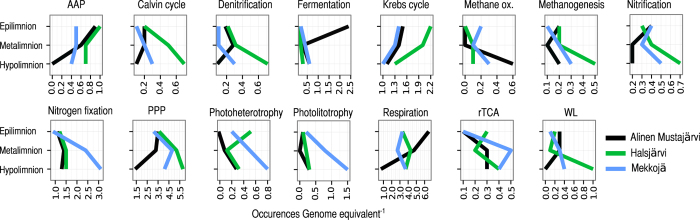
Depth distribution of sequences related to key pathways between the three layers of Alinen Mustajärvi, Halsjärvi and Mekkojärvi. X-axis values specify how many times on average each marker was found in one genome equivalent. AAP = Aerobic Anoxygenic Phototrophy, methane ox. = methane oxidation, PPP = Pentose Phosphate Pathway, rTCA = reverse Kreps Cycle, WL = Wood-Ljungdahl cycle. Note differences in x-axis scales.

Consistently, the frequency of autotrophic carbon fixation markers was increasing towards the bottom of the lakes, i.e. Calvin cycle, reverse tricarboxylic acid cycle (rTCA) and Wood-Ljungdahl pathway (WL) (Fig. 3). In the surface layer, the most likely group of organisms driving carbon fixation are *Cyanobacteria* and eukaryotic algae performing oxygenic photosynthesis. Also genes representing aerobic anoxygenic photosynthesis (AAP) were detected, with highest representation in the surface layer, an observation that agrees with previous findings of AAP bacteria in the epilimnia of humic lakes^16^ and their known role as strict aerobes^17^. While the proportion of *Cyanobacteria* remained relatively constant throughout the water column, some other known phototrophs affiliated with *Chloroflexi* were present only in the deeper water layers. This group is known to harbor Calvin cycle in their genome, thus explaining the increase in the potential for Calvin cycle^18^. The strongest candidate for hosting the rTCA cycle in the hypolimnion was *Chlorobium*, representing a phototrophic organism that is strictly anaerobic. To what extent the implied carbon fixation potentials are realized in the hypolimnion is not entirely clear, as both *Chloroflexi* and *Chlorobium* have the ability to grow organoheterotrophically^19^,^20^.

The third carbon fixation strategy, the WL pathway, is used to incorporate inorganic carbon into biomass in anoxic environments and is for example found in methanogens^21^. The frequency of methanogenesis gene markers and also methanotrophy gene markers was somewhat surprisingly highest in the hypolimnetic waters. In a previous report the highest methane oxidation rates in a humic lake was located at the oxygen transition zone^3^. To our knowledge there are so far no reports of anaerobic methane oxidation from the water column of humic lakes. However, isotopic measurements clearly indicate ongoing methanotrophic activity in the hypolimnion of Alinen Mustajärvi^11^ suggesting that our metagenomic predictions are realized also at the process level. The most abundant methanogen was *Methanoregula*, and most of the methane oxidizers belonged to type I methanotrophs with *Methanococcales* sp. as the most abundant taxon.

The frequency of markers for nitrogen transformations, such as amo, nif , nir, and nos genes, also increased towards the bottom (Fig. 3). Taxonomic annotations showed that taxa related to *Beta*- and *Gammaproteobacteria* were contributing to nitrification as well as denitrification. More specifically, we could link amo (nitrification) genes to the family *Burkholderiaceae*, whereas nir (denitrification) genes were related to families *Methylococcaceae* and *Sulfuricellaceae* and nos genes to the family *Sphingobacteriaceae (Bacteriodetes)*. Within the denitrification pathway, the ratio between the genes indicative for N_2_O production and consumption suggested net N_2_O production (Fig. S1). This is in agreement with a recent study reporting net N_2_O accumulation in boreal lakes^2^. The most prominent phylum capable of nitrogen fixation (nif) was *Chlorobia* (family *Chlorobiaceae*), especially in the hypolimnion. Even though nutrient concentrations in the hypolimnion are high, recent observations from marine systems have shown that active nitrogen fixation may occur even under nutrient-rich and dark conditions^22^.

To conclude, stratified humic lakes present a unique lake environment where different metabolic pathways and organisms are separated and enriched in response to changing availability of nutrients and electron acceptors. While these lakes represent a source of GHG emissions, they also harbor high genetic potential for carbon and nitrogen assimilation in their deep anoxic layers.

## Methods

Samples were collected from Alinen Mustajärvi (61°12'N, 25°06'E), Halsjärvi (61°13'N 25°08'E) and Mekkojärvi (61°13'N, 25°08'E) during summer stratification. Water was taken along a vertical profile at 0.5–1 m intervals using a Limnos water sampler (height: 30 cm, volume: 2.1 l). The collected water was then first passed through a 50-μm mesh to remove larger zooplankton. The samples were subsequently pooled according to oxygen concentration and temperature representing oxic epilimnion (oxygen > 1 mg L^−1^, stable temperature), oxygen transition zone (metalimnion; sharp decline in oxygen and temperature) and suboxic/anoxic hypolimnion (oxygen < 0.5 mg L^−1^, stable temperature) (see Table 1 for detail). The pooled water samples (volume 8 L) were transported to the laboratory within 2 hours of collection at ambient air temperature (+15 °C), subsequently stored at +4 °C and processed by tangential flow filtration (Durapore cassette, pore size 0.22 mm; Millipore, Billerica, MA, USA) within 4 hours of collection. Concentrated particles retained in the resulting ultrafiltration were frozen and freeze dried with an Alpha 1-4 LD plus (Christ, Osterode, Germany). For background information, pH, concentrations of NO_2_/NO_3_, NH_4_, PO_4_, total N, total P, dissolved organic carbon and chlorophyll *a* were measured from the pooled samples. In addition, CO_2_ and CH_4_ gas concentrations were measured at 1 m intervals while the O_2_ concentration and temperature were measured at 0.5 m intervals across the vertical profile. All measurements were done as described in Peura *et al*.^5^.

DNA from the nine samples of the three humic lakes was extracted from freeze-dried water samples using MOBIO PowerSoil DNA isolation kits (MO BIO Laboratories). Since DNA concentrations were too low for library preparation, 10 ng of DNA from each sample were amplified by multi displacement amplification using phi29 DNA polymerase (Thermo Scientific). 454 pyrosequencing was performed by the SNP & SEQ platform of the Science for Life Laboratory at Uppsala University using a Life Science 454 GS Titanium pyrosequencer with standard Titanium chemistry. The metagenomes have been deposited in SRA under accession number SRP052712. Raw sequence files of epilimnetic metagenomes from another 11 lakes (ten clearwater lakes and one humic) were taken from the study by Eiler *et al*.^9^. All datasets were subject to the same preprocessing criteria including length (length > 150) and quality filtering (mean quality > 21), and clustering artificial duplicates with cd-hit-454^23^. The hypolimnetic sample from Alinen Mustajärvi revealed a viral bloom as 64% of the reads matched viral entries in the NCBI-nr database. Thus, the sequencing depth for bacteria in this sample was very low and this was considered in the interpretation of data.

The processed sequences from humic lakes as well as those from clearwater lake metagenomes were searched against the STRING database^10^ using the BLASTX algorithm (max_targets 10, -evalue 0.001, -seg yes). Proteins in this database are grouped into clusters of orthologous groups (COG)^24^ and the results from the similarity search were then normalized using the frequency of 35 essential and single copy COGs^25^,^26^.

Normalized COG abundances were used to test the differences in functional diversity between the epilimnetic samples of the two lake types using PERMANOVA. In addition, the differences in functional potential between different layers of humic lakes were tested from COGs using PERMANOVA and visualized by using non-metric multidimentional scaling (NMDS). In both tests, one epilimnetic metagenome from humic lake recently published by Eiler *et al*.^9^ was included alongside the humic lake pool. Furthermore, the abundances of individual COGs in the epilimnetic humic lake metagenomes were compared to those of clear water lake metagenomes using a Wilcoxon rank sum test.

The potential for key pathways in carbon and nitrogen cycle was assessed using HMM models retrieved from the Pfam database using Hmmer3^27^ (hmmer.org; hmmsearch with default settings). The list of used Pfams is in Table S2. The occurrences of the Pfams in each metagenome were normalized using the occurrences of 139 single copy genes^15^. For pathways where multiple Pfams were available, the average occurrence was calculated. The differences in functional potential were visualized using NMDS plots including the loadings of individual Pfams. The taxonomic distribution in each sample was evaluated by inputing the result of a BLAST search (blastx, -evalue 0.001, -seg yes) against NCBI’s nr database into the MEGAN software^28^. All statistical analyses were conducted using R^29^ (http://www. R-project.org/). All the rest of the code produced for processing the sequencing data was written in Python and is available at https://github.com/xapple/humic.

## Additional Information

**How to cite this article**: Peura, S. *et al*. Metagenomic insights into strategies of aerobic and anaerobic carbon and nitrogen transformation in boreal lakes. *Sci. Rep*. **5**, 12102; doi: 10.1038/srep12102 (2015).

## Supplementary Material

Supplementary Information

## Figures and Tables

**Table 1:** General characteristics of the lakes and metagenomic data. Genome equivalents were calculated based on average number of 139 bacterial single copy genes in each sample.

	Alinen Mustajärvi			Halsjärvi			Mekkojärvi		
Feature	Epi	Meta	Hypo	Epi	Meta	Hypo	Epi	Meta	Hypo
Depth (m)	2	1	3.5	2	2	3.5	1	1	1.8
Temperature (C°)	16.3	10.3	4.9	14.5	7.2	5.0	13.1	7.9	5.3
Oxygen (mg l^−1^)	8.5	0.6	0.2	6.8	0.5	0.5	4.3	0.5	0.5
pH	5.1	4.8	5.7	6.4	6.2	6.4	5.1	5.4	5.9
DOC (mg l^−1^)	11.4	12.8	16.9	8.9	10.6	14.7	25.4	26.9	27.4
Chlorophyll (μg l^−1^)	7.2	36.6	83.6	9.9	28.1	34.4	9.3	50.1	63.1
CH_4_ (μM)	1.4	4	1100	0.5	99	370	2.5	37	200
Total N (μg l^−1^)	412	438	1880	370	440	1040	600	760	1750
Total P (μg l^−1^)	14	20	94	8	13	21	14	37	161
NO_2_ + NO_3_ (μg l^−1^)	15	11	11	20	35	61	41	40	48
NH_4_ (μg l^−1^)	8	7	1500	13	155	590	13	110	790
PO_4_ (μg l^−1^)	2	2	52	3	3	4	2	12	123
Data size (Mb)	69.5	64.6	150.5	30.0	43.0	22.1	43.4	30.2	27.0
Genome equivalents	10.0	11.8	3.5	22.3	18.1	9.0	27.8	15.3	13.5
