# Supplementary material for: Metagenomic insights into strategies of aerobic and anaerobic carbon and nitrogen transformation in boreal lakes
Source: Sci Rep. 2015 Jul 10;5:12102. doi: 10.1038/srep12102 (PMC4498382; doi:10.1038/srep12102)
Supplement: Supplementary Information [file srep12102-s1.pdf]

**Metagenomic insights into strategies of aerobic and anaerobic carbon and nitrogen transformation in boreal lakes**

Sari Peura, Lucas Sinclair, Stefan Bertilsson, Alexander Eiler

**Supplementary information.**

Supplementary Figure S1. Normalized occurrence of Nir and Nos genes in the lakes.

Supplementary Table S1. Results from a Wilcoxon rank sum test displaying the significant differences between the occurrences of COGs in the epilimnia of humic and clear water lakes.

Supplementary Table S2. Protein families (Pfams) used as markers for key metabolic pathways.

Supplementary Figure S1

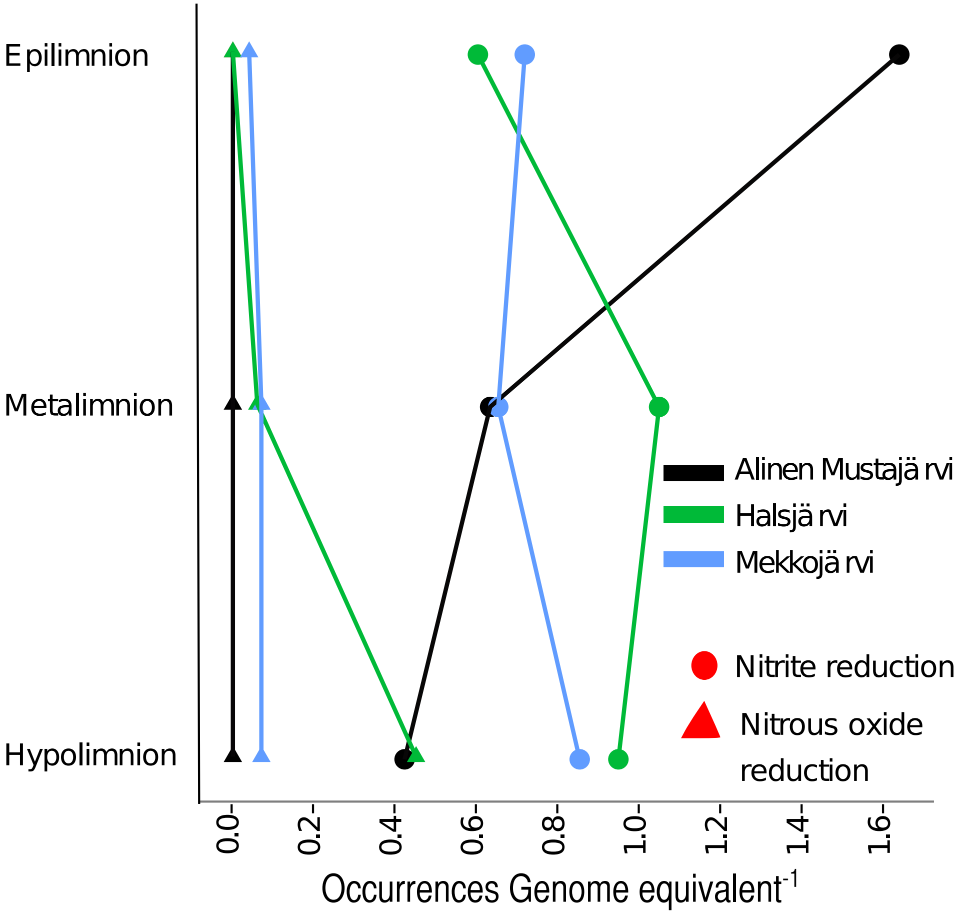

## Supplementary Table S1

| COG number | Enzyme name | overrepresented in | p-value |
|---|---|---|---|
| COG0004 | Ammonia permease | humic | 0.0091 |
| COG0007 | Uroporphyrinogen-III methylase | humic | 0.0046 |
| COG0017 | Aspartyl/asparaginyl-tRNA synthetases | humic | 0.0413 |
| COG0029 | Aspartate oxidase | humic | 0.0173 |
| COG0034 | Glutamine phosphoribosylpyrophosphate amidotransferase | humic | 0.0126 |
| COG0038 | Chloride channel protein EriC | humic | 0.0065 |
| COG0040 | ATP phosphoribosyltransferase | humic | 0.0032 |
| COG0063 | Predicted sugar kinase | humic | 0.0046 |
| COG0068 | Hydrogenase maturation factor | humic | 0.0416 |
| COG0069 | Glutamate synthase domain 2 | humic | 0.0312 |
| COG0071 | Molecular chaperone (small heat shock protein) | humic | 0.0173 |
| COG0073 | EMAP domain | humic | 0.0091 |
| COG0077 | Prephenate dehydratase | clear water | 0.0234 |
| COG0079 | Histidinol-phosphate/aromatic aminotransferase and cobyric acid decarboxylase | humic | 0.0032 |
| COG0088 | Ribosomal protein L4 | clear water | 0.0173 |
| COG0091 | Ribosomal protein L22 | clear water | 0.0173 |
| COG0092 | Ribosomal protein S3 | clear water | 0.0173 |
| COG0100 | Ribosomal protein S11 | humic | 0.0413 |
| COG0110 | Acetyltransferase (isoleucine patch superfamily) | humic | 0.0032 |
| COG0113 | Delta-aminolevulinic acid dehydratase | humic | 0.0046 |
| COG0116 | Predicted N6-adenine-specific DNA methylase | humic | 0.0091 |
| COG0120 | Ribose 5-phosphate isomerase | humic | 0.0046 |
| COG0131 | Imidazoleglycerol-phosphate dehydratase | clear water | 0.0234 |
| COG0132 | Dethiobiotin synthetase | humic | 0.0173 |
| COG0134 | Indole-3-glycerol phosphate synthase | humic | 0.0022 |
| COG0137 | Argininosuccinate synthase | humic | 0.0091 |
| COG0140 | Phosphoribosyl-ATP pyrophosphohydrolase | humic | 0.0413 |
| COG0147 | Anthranilate/para-aminobenzoate synthases component I | humic | 0.0046 |
| COG0151 | Phosphoribosylamine-glycine ligase | humic | 0.0022 |
| COG0152 | Phosphoribosylaminoimidazolesuccinocarboxamide (SAICAR) synthase | humic | 0.0022 |
| COG0156 | 7-keto-8-aminopelargonate synthetase and related enzymes | humic | 0.0091 |
| COG0161 | Adenosylmethionine-8-amino-7-oxononanoate aminotransferase | humic | 0.0091 |
| COG0164 | Ribonuclease HII | humic | 0.0312 |
| COG0173 | Aspartyl-tRNA synthetase | humic | 0.0046 |
| COG0174 | Glutamine synthetase | clear water | 0.0173 |
| COG0189 | Glutathione synthase/Ribosomal protein S6 modification enzyme (glutaminyl transferase) | humic | 0.0022 |
| COG0194 | Guanylate kinase | humic | 0.0022 |
| COG0206 | Cell division GTPase | humic | 0.0091 |
| COG0207 | Thymidylate synthase | humic | 0.0413 |
| COG0213 | Thymidine phosphorylase | clear water | 0.0126 |
| COG0214 | Pyridoxine biosynthesis enzyme | clear water | 0.0173 |
| COG0218 | Predicted GTPase | humic | 0.0126 |

| | | | |
|---|---|---|---|
| COG0221 | Inorganic pyrophosphatase | humic | 0.0312 |
| COG0234 | Co-chaperonin GroES (HSP10) | humic | 0.0032 |
| COG0241 | Histidinol phosphatase and related phosphatases | humic | 0.0065 |
| COG0242 | N-formylmethionyl-tRNA deformylase | humic | 0.0312 |
| COG0256 | Ribosomal protein L18 | clear water | 0.0312 |
| COG0261 | Ribosomal protein L21 | humic | 0.0413 |
| COG0262 | Dihydrofolate reductase | humic | 0.0022 |
| COG0270 | Site-specific DNA methylase | humic | 0.0091 |
| COG0272 | NAD-dependent DNA ligase (contains BRCT domain type II) | humic | 0.0022 |
| COG0276 | Protoheme ferro-lyase (ferrochelatase) | humic | 0.0091 |
| COG0277 | FAD/FMN-containing dehydrogenases | humic | 0.0091 |
| COG0282 | Acetate kinase | humic | 0.0456 |
| COG0284 | Orotidine-5'-phosphate decarboxylase | humic | 0.0022 |
| COG0286 | Type I restriction-modification system methyltransferase subunit | humic | 0.0046 |
| COG0305 | Replicative DNA helicase | humic | 0.0413 |
| COG0306 | Phosphate/sulphate permeases | humic | 0.0312 |
| COG0311 | Predicted glutamine amidotransferase involved in pyridoxine biosynthesis | clear water | 0.0107 |
| COG0315 | Molybdenum cofactor biosynthesis enzyme | humic | 0.0091 |
| COG0316 | Uncharacterized conserved protein | humic | 0.0091 |
| COG0324 | tRNA delta(2)-isopentenylpyrophosphate transferase | humic | 0.0234 |
| COG0332 | 3-oxoacyl-[acyl-carrier-protein] synthase III | humic | 0.0091 |
| COG0340 | Biotin-(acetyl-CoA carboxylase) ligase | humic | 0.0413 |
| COG0344 | Predicted membrane protein | humic | 0.0413 |
| COG0345 | Pyrroline-5-carboxylate reductase | humic | 0.0022 |
| COG0347 | Nitrogen regulatory protein PII | humic | 0.0413 |
| COG0348 | Polyferredoxin | humic | 0.0091 |
| COG0351 | Hydroxymethylpyrimidine/phosphomethylpyrimidine kinase | humic | 0.0022 |
| COG0352 | Thiamine monophosphate synthase | humic | 0.0126 |
| COG0361 | Translation initiation factor 1 (IF-1) | humic | 0.0126 |
| COG0362 | 6-phosphogluconate dehydrogenase | humic | 0.0065 |
| COG0370 | Fe2+ transport system protein B | humic | 0.0022 |
| COG0371 | Glycerol dehydrogenase and related enzymes | clear water | 0.0289 |
| COG0373 | Glutamyl-tRNA reductase | humic | 0.0126 |
| COG0375 | Zn finger protein HypA/HybF (possibly regulating hydrogenase expression) | humic | 0.0442 |
| COG0376 | Catalase (peroxidase I) | humic | 0.0091 |
| COG0378 | Ni2+-binding GTPase involved in regulation of expression and maturation of urease and hydrogenase | humic | 0.0413 |
| COG0379 | Quinolinate synthase | humic | 0.0234 |
| COG0381 | UDP-N-acetylglucosamine 2-epimerase | humic | 0.0022 |
| COG0382 | 4-hydroxybenzoate polyprenyltransferase and related prenyltransferases | humic | 0.0065 |
| COG0387 | Ca2+/H+ antiporter | humic | 0.0091 |
| COG0391 | Uncharacterized conserved protein | clear water | 0.0413 |
| COG0399 | Predicted pyridoxal phosphate-dependent enzyme apparently involved in regulation of cell wall biogenesis | humic | 0.0046 |
| COG0400 | Predicted esterase | humic | 0.0126 |
| COG0404 | Glycine cleavage system T protein (aminomethyltransferase) | clear water | 0.0312 |

| | | | |
|---|---|---|---|
| COG0407 | Uroporphyrinogen-III decarboxylase | humic | 0.0022 |
| COG0412 | Dienelactone hydrolase and related enzymes | humic | 0.0022 |
| COG0416 | Fatty acid/phospholipid biosynthesis enzyme | humic | 0.0126 |
| COG0417 | DNA polymerase elongation subunit (family B) | humic | 0.0234 |
| COG0422 | Thiamine biosynthesis protein ThiC | humic | 0.0312 |
| COG0426 | Uncharacterized flavoproteins | humic | 0.0182 |
| COG0427 | Acetyl-CoA hydrolase | humic | 0.0065 |
| COG0433 | Predicted ATPase | humic | 0.0312 |
| COG0438 | Glycosyltransferase | humic | 0.0312 |
| COG0443 | Molecular chaperone | humic | 0.0312 |
| COG0445 | NAD/FAD-utilizing enzyme apparently involved in cell division | humic | 0.0022 |
| COG0446 | Uncharacterized NAD(FAD)-dependent dehydrogenases | humic | 0.0234 |
| COG0450 | Peroxiredoxin | humic | 0.0173 |
| COG0451 | Nucleoside-diphosphate-sugar epimerases | humic | 0.0173 |
| COG0457 | FOG: TPR repeat | humic | 0.0234 |
| COG0463 | Glycosyltransferases involved in cell wall biogenesis | humic | 0.0091 |
| COG0464 | ATPases of the AAA+ class | humic | 0.0126 |
| COG0468 | RecA/RadA recombinase | humic | 0.0022 |
| COG0470 | ATPase involved in DNA replication | humic | 0.0234 |
| COG0473 | Isocitrate/isopropylmalate dehydrogenase | humic | 0.0091 |
| COG0474 | Cation transport ATPase | humic | 0.0065 |
| COG0476 | Dinucleotide-utilizing enzymes involved in molybdopterin and thiamine biosynthesis family 2 | humic | 0.0065 |
| COG0477 | Permeases of the major facilitator superfamily | humic | 0.0022 |
| COG0481 | Membrane GTPase LepA | humic | 0.0312 |
| COG0486 | Predicted GTPase | humic | 0.0022 |
| COG0489 | ATPases involved in chromosome partitioning | humic | 0.0022 |
| COG0492 | Thioredoxin reductase | humic | 0.0091 |
| COG0494 | NTP pyrophosphohydrolases including oxidative damage repair enzymes | humic | 0.0126 |
| COG0497 | ATPase involved in DNA repair | humic | 0.0046 |
| COG0501 | Zn-dependent protease with chaperone function | humic | 0.0413 |
| COG0502 | Biotin synthase and related enzymes | humic | 0.0234 |
| COG0510 | Predicted choline kinase involved in LPS biosynthesis | clear water | 0.0413 |
| COG0514 | Superfamily II DNA helicase | humic | 0.0126 |
| COG0521 | Molybdopterin biosynthesis enzymes | humic | 0.0173 |
| COG0525 | Valyl-tRNA synthetase | humic | 0.0234 |
| COG0526 | Thiol-disulfide isomerase and thioredoxins | humic | 0.0091 |
| COG0531 | Amino acid transporters | humic | 0.0046 |
| COG0535 | Predicted Fe-S oxidoreductases | humic | 0.0173 |
| COG0543 | 2-polyprenylphenol hydroxylase and related flavodoxin oxidoreductases | humic | 0.0091 |
| COG0550 | Topoisomerase IA | humic | 0.0173 |
| COG0551 | Zn-finger domain associated with topoisomerase type I | humic | 0.0046 |
| COG0556 | Helicase subunit of the DNA excision repair complex | humic | 0.0312 |
| COG0561 | Predicted hydrolases of the HAD superfamily | clear water | 0.0413 |
| COG0562 | UDP-galactopyranose mutase | clear water | 0.0312 |

| | | | |
|---|---|---|---|
| COG0563 | Adenylate kinase and related kinases | humic | 0.0312 |
| COG0565 | rRNA methylase | humic | 0.0173 |
| COG0572 | Uridine kinase | clear water | 0.0385 |
| COG0575 | CDP-diglyceride synthetase | humic | 0.0234 |
| COG0582 | Integrase | humic | 0.0032 |
| COG0589 | Universal stress protein UspA and related nucleotide-binding proteins | humic | 0.0234 |
| COG0590 | Cytosine/adenosine deaminases | humic | 0.0032 |
| COG0591 | Na+/proline symporter | humic | 0.0022 |
| COG0592 | DNA polymerase sliding clamp subunit (PCNA homolog) | humic | 0.0022 |
| COG0594 | RNase P protein component | humic | 0.0312 |
| COG0599 | Uncharacterized homolog of gamma-carboxymuconolactone decarboxylase subunit | humic | 0.0126 |
| COG0602 | Organic radical activating enzymes | humic | 0.0022 |
| COG0608 | Single-stranded DNA-specific exonuclease | humic | 0.0065 |
| COG0611 | Thiamine monophosphate kinase | humic | 0.0022 |
| COG0612 | Predicted Zn-dependent peptidases | humic | 0.0173 |
| COG0629 | Single-stranded DNA-binding protein | humic | 0.0022 |
| COG0635 | Coproporphyrinogen III oxidase and related Fe-S oxidoreductases | humic | 0.0234 |
| COG0640 | Predicted transcriptional regulators | humic | 0.0234 |
| COG0641 | Arylsulfatase regulator (Fe-S oxidoreductase) | humic | 0.0411 |
| COG0642 | Signal transduction histidine kinase | humic | 0.0046 |
| COG0644 | Dehydrogenases (flavoproteins) | humic | 0.0234 |
| COG0655 | Multimeric flavodoxin WrbA | humic | 0.0173 |
| COG0664 | cAMP-binding proteins - catabolite gene activator and regulatory subunit of cAMP-dependent protein kinases | humic | 0.0234 |
| COG0665 | Glycine/D-amino acid oxidases (deaminating) | clear water | 0.0046 |
| COG0671 | Membrane-associated phospholipid phosphatase | humic | 0.0173 |
| COG0675 | Transposase and inactivated derivatives | humic | 0.0312 |
| COG0682 | Prolipoprotein diacylglyceryltransferase | humic | 0.0032 |
| COG0686 | Alanine dehydrogenase | humic | 0.0032 |
| COG0687 | Spermidine/putrescine-binding periplasmic protein | clear water | 0.0046 |
| COG0689 | RNase PH | clear water | 0.0173 |
| COG0701 | Predicted permeases | humic | 0.0046 |
| COG0707 | UDP-N-acetylglucosamine:LPS N-acetylglucosamine transferase | humic | 0.0022 |
| COG0708 | Exonuclease III | humic | 0.0022 |
| COG0709 | Selenophosphate synthase | humic | 0.0065 |
| COG0720 | 6-pyruvoyl-tetrahydropterin synthase | humic | 0.0022 |
| COG0724 | RNA-binding proteins (RRM domain) | humic | 0.0126 |
| COG0726 | Predicted xylanase/chitin deacetylase | humic | 0.0022 |
| COG0732 | Restriction endonuclease S subunits | humic | 0.0173 |
| COG0735 | Fe2+/Zn2+ uptake regulation proteins | clear water | 0.0413 |
| COG0736 | Phosphopantetheinyl transferase (holo-ACP synthase) | humic | 0.0022 |
| COG0738 | Fucose permease | humic | 0.0065 |
| COG0739 | Membrane proteins related to metalloendopeptidases | humic | 0.0234 |
| COG0741 | Soluble lytic murein transglycosylase and related regulatory proteins (some contain LysM/invasin domains) | humic | 0.0022 |
| COG0743 | 1-deoxy-D-xylulose 5-phosphate reductoisomerase | humic | 0.0022 |

| | | | |
|---|---|---|---|
| COG0745 | Response regulators consisting of a CheY-like receiver domain and a winged-helix DNA-binding domain | humic | 0.0234 |
| COG0746 | Molybdopterin-guanine dinucleotide biosynthesis protein A | humic | 0.0046 |
| COG0749 | DNA polymerase I - 3'-5' exonuclease and polymerase domains | humic | 0.0065 |
| COG0758 | Predicted Rossmann fold nucleotide-binding protein involved in DNA uptake | humic | 0.0234 |
| COG0760 | Parvulin-like peptidyl-prolyl isomerase | humic | 0.0173 |
| COG0762 | Predicted integral membrane protein | humic | 0.0413 |
| COG0768 | Cell division protein FtsI/penicillin-binding protein 2 | humic | 0.0022 |
| COG0773 | UDP-N-acetylmuramate-alanine ligase | humic | 0.0312 |
| COG0774 | UDP-3-O-acyl-N-acetylglucosamine deacetylase | humic | 0.0312 |
| COG0776 | Bacterial nucleoid DNA-binding protein | humic | 0.0173 |
| COG0782 | Transcription elongation factor | humic | 0.0312 |
| COG0783 | DNA-binding ferritin-like protein (oxidative damage protectant) | humic | 0.0413 |
| COG0784 | FOG: CheY-like receiver | humic | 0.0065 |
| COG0786 | Na+/glutamate symporter | clear water | 0.0183 |
| COG0788 | Formyltetrahydrofolate hydrolase | humic | 0.0091 |
| COG0793 | Periplasmic protease | humic | 0.0091 |
| COG0794 | Predicted sugar phosphate isomerase involved in capsule formation | humic | 0.0312 |
| COG0795 | Predicted permeases | humic | 0.0173 |
| COG0796 | Glutamate racemase | humic | 0.0312 |
| COG0798 | Arsenite efflux pump ACR3 and related permeases | humic | 0.0022 |
| COG0802 | Predicted ATPase or kinase | humic | 0.0046 |
| COG0805 | Sec-independent protein secretion pathway component TatC | humic | 0.0065 |
| COG0809 | S-adenosylmethionine:tRNA-ribosyltransferase-isomerase (queuine synthetase) | humic | 0.0234 |
| COG0811 | Biopolymer transport proteins | humic | 0.0312 |
| COG0813 | Purine-nucleoside phosphorylase | clear water | 0.0062 |
| COG0823 | Periplasmic component of the Tol biopolymer transport system | humic | 0.0126 |
| COG0825 | Acetyl-CoA carboxylase alpha subunit | humic | 0.0126 |
| COG0826 | Collagenase and related proteases | humic | 0.0022 |
| COG0827 | Adenine-specific DNA methylase | humic | 0.0312 |
| COG0831 | Urea amidohydrolase (urease) gamma subunit | clear water | 0.0093 |
| COG0832 | Urea amidohydrolase (urease) beta subunit | humic | 0.0172 |
| COG0836 | Mannose-1-phosphate guanylyltransferase | humic | 0.0173 |
| COG0837 | Glucokinase | clear water | 0.0093 |
| COG0841 | Cation/multidrug efflux pump | humic | 0.0022 |
| COG0845 | Membrane-fusion protein | humic | 0.0091 |
| COG0848 | Biopolymer transport protein | humic | 0.0046 |
| COG0849 | Actin-like ATPase involved in cell division | humic | 0.0022 |
| COG0852 | NADH:ubiquinone oxidoreductase 27 kD subunit | humic | 0.0173 |
| COG0853 | Aspartate 1-decarboxylase | humic | 0.0172 |
| COG0858 | Ribosome-binding factor A | clear water | 0.0173 |
| COG0859 | ADP-heptose:LPS heptosyltransferase | humic | 0.0022 |
| COG0860 | N-acetylmuramoyl-L-alanine amidase | humic | 0.0413 |
| COG0863 | DNA modification methylase | humic | 0.0234 |
| COG1001 | Adenine deaminase | humic | 0.0126 |

| | | | |
|---|---|---|---|
| COG1005 | NADH:ubiquinone oxidoreductase subunit 1 (chain H) | humic | 0.0022 |
| COG1011 | Predicted hydrolase (HAD superfamily) | humic | 0.0065 |
| COG1029 | Formylmethanofuran dehydrogenase subunit B | humic | 0.0011 |
| COG1032 | Fe-S oxidoreductase | humic | 0.0234 |
| COG1034 | NADH dehydrogenase/NADH:ubiquinone oxidoreductase 75 kD subunit (chain G) | humic | 0.0173 |
| COG1044 | UDP-3-O-[3-hydroxymyristoyl] glucosamine N-acyltransferase | humic | 0.0022 |
| COG1047 | FKBP-type peptidyl-prolyl cis-trans isomerases 2 | humic | 0.0126 |
| COG1049 | Aconitase B | humic | 0.0234 |
| COG1051 | ADP-ribose pyrophosphatase | humic | 0.0046 |
| COG1052 | Lactate dehydrogenase and related dehydrogenases | humic | 0.0173 |
| COG1056 | Nicotinamide mononucleotide adenylyltransferase | humic | 0.0030 |
| COG1061 | DNA or RNA helicases of superfamily II | humic | 0.0065 |
| COG1063 | Threonine dehydrogenase and related Zn-dependent dehydrogenases | humic | 0.0312 |
| COG1064 | Zn-dependent alcohol dehydrogenases | humic | 0.0022 |
| COG1067 | Predicted ATP-dependent protease | humic | 0.0046 |
| COG1074 | ATP-dependent exoDNAse (exonuclease V) beta subunit (contains helicase and exonuclease domains) | humic | 0.0046 |
| COG1083 | CMP-N-acetylneuraminic acid synthetase | clear water | 0.0045 |
| COG1113 | Gamma-aminobutyrate permease and related permeases | humic | 0.0046 |
| COG1138 | Cytochrome c biogenesis factor | humic | 0.0046 |
| COG1145 | Ferredoxin | humic | 0.0046 |
| COG1146 | Ferredoxin | clear water | 0.0234 |
| COG1179 | Dinucleotide-utilizing enzymes involved in molybdopterin and thiamine biosynthesis family 1 | humic | 0.0091 |
| COG1180 | Pyruvate-formate lyase-activating enzyme | humic | 0.0313 |
| COG1183 | Phosphatidylserine synthase | humic | 0.0046 |
| COG1187 | 16S rRNA uridine-516 pseudouridylate synthase and related pseudouridylate synthases | humic | 0.0173 |
| COG1191 | DNA-directed RNA polymerase specialized sigma subunit | clear water | 0.0173 |
| COG1192 | ATPases involved in chromosome partitioning | humic | 0.0413 |
| COG1199 | Rad3-related DNA helicases | clear water | 0.0312 |
| COG1209 | dTDP-glucose pyrophosphorylase | humic | 0.0022 |
| COG1210 | UDP-glucose pyrophosphorylase | humic | 0.0065 |
| COG1230 | Co/Zn/Cd efflux system component | humic | 0.0046 |
| COG1233 | Phytoene dehydrogenase and related proteins | clear water | 0.0413 |
| COG1235 | Metal-dependent hydrolases of the beta-lactamase superfamily I | humic | 0.0065 |
| COG1236 | Predicted exonuclease of the beta-lactamase fold involved in RNA processing | humic | 0.0065 |
| COG1239 | Mg-chelatase subunit ChlI | clear water | 0.0065 |
| COG1251 | NAD(P)H-nitrite reductase | humic | 0.0224 |
| COG1260 | Myo-inositol-1-phosphate synthase | clear water | 0.0234 |
| COG1266 | Predicted metal-dependent membrane protease | clear water | 0.0044 |
| COG1267 | Phosphatidylglycerophosphatase A and related proteins | humic | 0.0234 |
| COG1270 | Cobalamin biosynthesis protein CobD/CbiB | humic | 0.0173 |
| COG1274 | Phosphoenolpyruvate carboxykinase (GTP) | humic | 0.0032 |
| COG1276 | Putative copper export protein | clear water | 0.0338 |
| COG1296 | Predicted branched-chain amino acid permease (azaleucine resistance) | humic | 0.0312 |
| COG1301 | Na+/H+-dicarboxylate symporters | humic | 0.0022 |

| | | | |
|---|---|---|---|
| COG1310 | Predicted metal-dependent protease of the PAD1/JAB1 superfamily | clear water | 0.0383 |
| COG1314 | Preprotein translocase subunit SecG | clear water | 0.0338 |
| COG1322 | Uncharacterized protein conserved in bacteria | humic | 0.0032 |
| COG1328 | Oxygen-sensitive ribonucleoside-triphosphate reductase | humic | 0.0182 |
| COG1331 | Highly conserved protein containing a thioredoxin domain | humic | 0.0173 |
| COG1346 | Putative effector of murein hydrolase | humic | 0.0173 |
| COG1372 | Intein/homing endonuclease | humic | 0.0091 |
| COG1381 | Recombinational DNA repair protein (RecF pathway) | humic | 0.0234 |
| COG1386 | Predicted transcriptional regulator containing the HTH domain | humic | 0.0173 |
| COG1388 | FOG: LysM repeat | clear water | 0.0093 |
| COG1392 | Phosphate transport regulator (distant homolog of PhoU) | humic | 0.0091 |
| COG1398 | Fatty-acid desaturase | humic | 0.0312 |
| COG1401 | GTPase subunit of restriction endonuclease | humic | 0.0091 |
| COG1404 | Subtilisin-like serine proteases | clear water | 0.0065 |
| COG1427 | Predicted periplasmic solute-binding protein | humic | 0.0413 |
| COG1428 | Deoxynucleoside kinases | humic | 0.0091 |
| COG1432 | Uncharacterized conserved protein | humic | 0.0234 |
| COG1433 | Uncharacterized conserved protein | humic | 0.0382 |
| COG1435 | Thymidine kinase | clear water | 0.0234 |
| COG1441 | O-succinylbenzoate synthase | humic | 0.0014 |
| COG1451 | Predicted metal-dependent hydrolase | humic | 0.0126 |
| COG1452 | Organic solvent tolerance protein OstA | humic | 0.0046 |
| COG1457 | Purine-cytosine permease and related proteins | humic | 0.0046 |
| COG1462 | Uncharacterized protein involved in formation of curli polymers | humic | 0.0312 |
| COG1468 | RecB family exonuclease | humic | 0.0245 |
| COG1469 | Uncharacterized conserved protein | humic | 0.0173 |
| COG1472 | Beta-glucosidase-related glycosidases | humic | 0.0032 |
| COG1475 | Predicted transcriptional regulators | humic | 0.0065 |
| COG1476 | Predicted transcriptional regulators | clear water | 0.0043 |
| COG1477 | Membrane-associated lipoprotein involved in thiamine biosynthesis | humic | 0.0091 |
| COG1479 | Uncharacterized conserved protein | humic | 0.0126 |
| COG1482 | Phosphomannose isomerase | clear water | 0.0312 |
| COG1502 | Phosphatidylserine/phosphatidylglycerophosphate/cardiolipin synthases and related enzymes | humic | 0.0173 |
| COG1511 | Predicted membrane protein | clear water | 0.0183 |
| COG1519 | 3-deoxy-D-manno-octulosonic-acid transferase | humic | 0.0173 |
| COG1520 | FOG: WD40-like repeat | humic | 0.0091 |
| COG1525 | Micrococcal nuclease (thermonuclease) homologs | clear water | 0.0091 |
| COG1538 | Outer membrane protein | humic | 0.0022 |
| COG1546 | Uncharacterized protein (competence- and mitomycin-induced) | humic | 0.0173 |
| COG1554 | Trehalose and maltose hydrolases (possible phosphorylases) | humic | 0.0120 |
| COG1560 | Lauroyl/myristoyl acyltransferase | humic | 0.0091 |
| COG1561 | Uncharacterized stress-induced protein | humic | 0.0126 |
| COG1564 | Thiamine pyrophosphokinase | clear water | 0.0019 |
| COG1566 | Multidrug resistance efflux pump | humic | 0.0046 |

| | | | |
|---|---|---|---|
| COG1583 | Uncharacterized protein predicted to be involved in DNA repair (RAMP superfamily) | humic | 0.0382 |
| COG1592 | Rubrerythrin | humic | 0.0091 |
| COG1605 | Chorismate mutase | humic | 0.0312 |
| COG1606 | ATP-utilizing enzymes of the PP-loop superfamily | clear water | 0.0044 |
| COG1609 | Transcriptional regulators | humic | 0.0413 |
| COG1615 | Uncharacterized conserved protein | clear water | 0.0173 |
| COG1637 | Predicted nuclease of the RecB family | clear water | 0.0480 |
| COG1647 | Esterase/lipase | humic | 0.0312 |
| COG1648 | Siroheme synthase (precorrin-2 oxidase/ferrochelatase domain) | humic | 0.0413 |
| COG1650 | Uncharacterized protein conserved in archaea | humic | 0.0078 |
| COG1651 | Protein-disulfide isomerase | humic | 0.0065 |
| COG1663 | Tetraacyldisaccharide-1-P 4'-kinase | humic | 0.0022 |
| COG1664 | Integral membrane protein CcmA involved in cell shape determination | humic | 0.0022 |
| COG1666 | Uncharacterized protein conserved in bacteria | humic | 0.0065 |
| COG1672 | Predicted ATPase (AAA+ superfamily) | humic | 0.0173 |
| COG1674 | DNA segregation ATPase FtsK/SpoIIIE and related proteins | humic | 0.0173 |
| COG1678 | Putative transcriptional regulator | humic | 0.0091 |
| COG1686 | D-alanyl-D-alanine carboxypeptidase | humic | 0.0126 |
| COG1692 | Uncharacterized protein conserved in bacteria | humic | 0.0234 |
| COG1712 | Predicted dinucleotide-utilizing enzyme | clear water | 0.0093 |
| COG1714 | Predicted membrane protein/domain | humic | 0.0312 |
| COG1729 | Uncharacterized protein conserved in bacteria | humic | 0.0413 |
| COG1731 | Archaeal riboflavin synthase | humic | 0.0382 |
| COG1735 | Predicted metal-dependent hydrolase with the TIM-barrel fold | clear water | 0.0093 |
| COG1737 | Transcriptional regulators | clear water | 0.0030 |
| COG1738 | Uncharacterized conserved protein | humic | 0.0233 |
| COG1741 | Pirin-related protein | humic | 0.0022 |
| COG1752 | Predicted esterase of the alpha-beta hydrolase superfamily | humic | 0.0091 |
| COG1778 | Low specificity phosphatase (HAD superfamily) | humic | 0.0032 |
| COG1780 | Protein involved in ribonucleotide reduction | clear water | 0.0093 |
| COG1783 | Phage terminase large subunit | humic | 0.0065 |
| COG1785 | Alkaline phosphatase | humic | 0.0173 |
| COG1794 | Aspartate racemase | humic | 0.0046 |
| COG1807 | 4-amino-4-deoxy-L-arabinose transferase and related glycosyltransferases of PMT family | humic | 0.0312 |
| COG1815 | Flagellar basal body protein | clear water | 0.0363 |
| COG1825 | Ribosomal protein L25 (general stress protein Ctc) | humic | 0.0173 |
| COG1836 | Predicted membrane protein | clear water | 0.0183 |
| COG1872 | Uncharacterized conserved protein | humic | 0.0089 |
| COG1876 | D-alanyl-D-alanine carboxypeptidase | clear water | 0.0215 |
| COG1886 | Flagellar motor switch/type III secretory pathway protein | clear water | 0.0157 |
| COG1893 | Ketopantoate reductase | humic | 0.0312 |
| COG1910 | Periplasmic molybdate-binding protein/domain | humic | 0.0022 |
| COG1914 | Mn2+ and Fe2+ transporters of the NRAMP family | humic | 0.0022 |
| COG1921 | Selenocysteine synthase [seryl-tRNASer selenium transferase] | clear water | 0.0120 |

| | | | |
|---|---|---|---|
| COG1926 | Predicted phosphoribosyltransferases | clear water | 0.0338 |
| COG1952 | Preprotein translocase subunit SecB | humic | 0.0046 |
| COG1953 | Cytosine/uracil/thiamine/allantoin permeases | clear water | 0.0044 |
| COG1957 | Inosine-uridine nucleoside N-ribohydrolase | clear water | 0.0269 |
| COG1968 | Uncharacterized bacitracin resistance protein | humic | 0.0173 |
| COG1995 | Pyridoxal phosphate biosynthesis protein | humic | 0.0173 |
| COG2000 | Predicted Fe-S protein | humic | 0.0382 |
| COG2002 | Regulators of stationary/sporulation gene expression | clear water | 0.0338 |
| COG2003 | DNA repair proteins | humic | 0.0022 |
| COG2022 | Uncharacterized enzyme of thiazole biosynthesis | humic | 0.0046 |
| COG2033 | Desulfoferrodoxin | humic | 0.0382 |
| COG2039 | Pyrrolidone-carboxylate peptidase (N-terminal pyroglutamyl peptidase) | clear water | 0.0019 |
| COG2041 | Sulfite oxidase and related enzymes | humic | 0.0046 |
| COG2059 | Chromate transport protein ChrA | humic | 0.0126 |
| COG2062 | Phosphohistidine phosphatase SixA | humic | 0.0091 |
| COG2069 | CO dehydrogenase/acetyl-CoA synthase delta subunit (corrinoid Fe-S protein) | humic | 0.0382 |
| COG2072 | Predicted flavoprotein involved in K+ transport | clear water | 0.0234 |
| COG2073 | Cobalamin biosynthesis protein CbiG | clear water | 0.0338 |
| COG2076 | Membrane transporters of cations and cationic drugs | clear water | 0.0307 |
| COG2077 | Peroxiredoxin | humic | 0.0413 |
| COG2081 | Predicted flavoproteins | humic | 0.0065 |
| COG2082 | Precorrin isomerase | clear water | 0.0183 |
| COG2107 | Predicted periplasmic solute-binding protein | clear water | 0.0363 |
| COG2124 | Cytochrome P450 | clear water | 0.0065 |
| COG2152 | Predicted glycosylase | humic | 0.0065 |
| COG2161 | Antitoxin of toxin-antitoxin stability system | humic | 0.0173 |
| COG2162 | Arylamine N-acetyltransferase | humic | 0.0182 |
| COG2179 | Predicted hydrolase of the HAD superfamily | clear water | 0.0338 |
| COG2183 | Transcriptional accessory protein | humic | 0.0173 |
| COG2184 | Protein involved in cell division | humic | 0.0312 |
| COG2189 | Adenine specific DNA methylase Mod | humic | 0.0032 |
| COG2195 | Di- and tripeptidases | humic | 0.0091 |
| COG2197 | Response regulator containing a CheY-like receiver domain and an HTH DNA-binding domain | humic | 0.0065 |
| COG2199 | FOG: GGDEF domain | humic | 0.0046 |
| COG2200 | FOG: EAL domain | humic | 0.0413 |
| COG2202 | FOG: PAS/PAC domain | humic | 0.0046 |
| COG2203 | FOG: GAF domain | humic | 0.0413 |
| COG2205 | Osmosensitive K+ channel histidine kinase | humic | 0.0046 |
| COG2206 | HD-GYP domain | humic | 0.0022 |
| COG2210 | Uncharacterized conserved protein | humic | 0.0157 |
| COG2211 | Na+/melibiose symporter and related transporters | clear water | 0.0307 |
| COG2214 | DnaJ-class molecular chaperone | humic | 0.0046 |
| COG2217 | Cation transport ATPase | humic | 0.0173 |
| COG2226 | Methylase involved in ubiquinone/menaquinone biosynthesis | humic | 0.0312 |

| | | | |
|---|---|---|---|
| COG2230 | Cyclopropane fatty acid synthase and related methyltransferases | humic | 0.0046 |
| COG2231 | Uncharacterized protein related to Endonuclease III | humic | 0.0382 |
| COG2232 | Predicted ATP-dependent carboligase related to biotin carboxylase | humic | 0.0125 |
| COG2242 | Precorrin-6B methylase 2 | clear water | 0.0215 |
| COG2243 | Precorrin-2 methylase | clear water | 0.0338 |
| COG2244 | Membrane protein involved in the export of O-antigen and teichoic acid | humic | 0.0022 |
| COG2246 | Predicted membrane protein | clear water | 0.0093 |
| COG2256 | ATPase related to the helicase subunit of the Holliday junction resolvase | humic | 0.0046 |
| COG2258 | Uncharacterized protein conserved in bacteria | humic | 0.0065 |
| COG2264 | Ribosomal protein L11 methylase | humic | 0.0022 |
| COG2270 | Permeases of the major facilitator superfamily | clear water | 0.0413 |
| COG2273 | Beta-glucanase/Beta-glucan synthetase | humic | 0.0065 |
| COG2307 | Uncharacterized protein conserved in bacteria | humic | 0.0065 |
| COG2308 | Uncharacterized conserved protein | humic | 0.0022 |
| COG2311 | Predicted membrane protein | clear water | 0.0193 |
| COG2312 | Erythromycin esterase homolog | clear water | 0.0338 |
| COG2313 | Uncharacterized enzyme involved in pigment biosynthesis | clear water | 0.0046 |
| COG2321 | Predicted metalloprotease | clear water | 0.0183 |
| COG2326 | Uncharacterized conserved protein | humic | 0.0091 |
| COG2332 | Cytochrome c-type biogenesis protein CcmE | humic | 0.0126 |
| COG2333 | Predicted hydrolase (metallo-beta-lactamase superfamily) | humic | 0.0173 |
| COG2335 | Secreted and surface protein containing fasciclin-like repeats | clear water | 0.0172 |
| COG2346 | Truncated hemoglobins | humic | 0.0234 |
| COG2351 | Transthyretin-like protein | clear water | 0.0183 |
| COG2353 | Uncharacterized conserved protein | humic | 0.0126 |
| COG2354 | Uncharacterized protein conserved in bacteria | clear water | 0.0183 |
| COG2375 | Siderophore-interacting protein | humic | 0.0014 |
| COG2379 | Putative glycerate kinase | humic | 0.0234 |
| COG2391 | Predicted transporter component | humic | 0.0091 |
| COG2409 | Predicted drug exporters of the RND superfamily | clear water | 0.0234 |
| COG2421 | Predicted acetamidase/formamidase | humic | 0.0091 |
| COG2452 | Predicted site-specific integrase-resolvase | humic | 0.0230 |
| COG2453 | Predicted protein-tyrosine phosphatase | clear water | 0.0093 |
| COG2508 | Regulator of polyketide synthase expression | clear water | 0.0044 |
| COG2510 | Predicted membrane protein | humic | 0.0416 |
| COG2606 | Uncharacterized conserved protein | clear water | 0.0046 |
| COG2608 | Copper chaperone | clear water | 0.0338 |
| COG2706 | 3-carboxymuconate cyclase | humic | 0.0091 |
| COG2717 | Predicted membrane protein | humic | 0.0312 |
| COG2730 | Endoglucanase | humic | 0.0091 |
| COG2733 | Predicted membrane protein | clear water | 0.0385 |
| COG2740 | Predicted nucleic-acid-binding protein implicated in transcription termination | clear water | 0.0215 |
| COG2761 | Predicted dithiol-disulfide isomerase involved in polyketide biosynthesis | humic | 0.0126 |
| COG2764 | Uncharacterized protein conserved in bacteria | humic | 0.0313 |

| | | | |
|---|---|---|---|
| COG2768 | Uncharacterized Fe-S center protein | humic | 0.0254 |
| COG2801 | Transposase and inactivated derivatives | humic | 0.0032 |
| COG2808 | Transcriptional regulator | humic | 0.0032 |
| COG2813 | 16S RNA G1207 methylase RsmC | clear water | 0.0183 |
| COG2814 | Arabinose efflux permease | humic | 0.0022 |
| COG2824 | Uncharacterized Zn-ribbon-containing protein involved in phosphonate metabolism | humic | 0.0021 |
| COG2831 | Hemolysin activation/secretion protein | humic | 0.0046 |
| COG2833 | Uncharacterized protein conserved in bacteria | humic | 0.0022 |
| COG2836 | Uncharacterized conserved protein | humic | 0.0089 |
| COG2838 | Monomeric isocitrate dehydrogenase | humic | 0.0065 |
| COG2844 | UTP:GlnB (protein PII) uridylyltransferase | humic | 0.0022 |
| COG2845 | Uncharacterized protein conserved in bacteria | humic | 0.0113 |
| COG2850 | Uncharacterized conserved protein | humic | 0.0091 |
| COG2853 | Surface lipoprotein | humic | 0.0065 |
| COG2856 | Predicted Zn peptidase | humic | 0.0113 |
| COG2859 | Uncharacterized protein conserved in bacteria | clear water | 0.0413 |
| COG2860 | Predicted membrane protein | humic | 0.0091 |
| COG2862 | Predicted membrane protein | clear water | 0.0022 |
| COG2863 | Cytochrome c553 | humic | 0.0413 |
| COG2864 | Cytochrome b subunit of formate dehydrogenase | humic | 0.0065 |
| COG2865 | Predicted transcriptional regulator containing an HTH domain and an uncharacterized domain shared with the ma | humic | 0.0413 |
| COG2885 | Outer membrane protein and related peptidoglycan-associated (lipo)proteins | humic | 0.0022 |
| COG2890 | Methylase of polypeptide chain release factors | humic | 0.0022 |
| COG2891 | Cell shape-determining protein | humic | 0.0091 |
| COG2894 | Septum formation inhibitor-activating ATPase | clear water | 0.0385 |
| COG2896 | Molybdenum cofactor biosynthesis enzyme | humic | 0.0065 |
| COG2898 | Uncharacterized conserved protein | humic | 0.0046 |
| COG2907 | Predicted NAD/FAD-binding protein | humic | 0.0413 |
| COG2908 | Uncharacterized protein conserved in bacteria | humic | 0.0022 |
| COG2909 | ATP-dependent transcriptional regulator | humic | 0.0383 |
| COG2912 | Uncharacterized conserved protein | humic | 0.0413 |
| COG2913 | Small protein A (tmRNA-binding) | humic | 0.0173 |
| COG2924 | Fe-S cluster protector protein | humic | 0.0456 |
| COG2933 | Predicted SAM-dependent methyltransferase | clear water | 0.0093 |
| COG2935 | Putative arginyl-tRNA:protein arginylyltransferase | humic | 0.0173 |
| COG2944 | Predicted transcriptional regulator | humic | 0.0307 |
| COG2956 | Predicted N-acetylglucosaminyl transferase | humic | 0.0173 |
| COG2969 | Stringent starvation protein B | humic | 0.0021 |
| COG2976 | Uncharacterized protein conserved in bacteria | humic | 0.0126 |
| COG2978 | Putative p-aminobenzoyl-glutamate transporter | clear water | 0.0093 |
| COG2980 | Rare lipoprotein B | humic | 0.0065 |
| COG2982 | Uncharacterized protein involved in outer membrane biogenesis | humic | 0.0032 |
| COG2989 | Uncharacterized protein conserved in bacteria | humic | 0.0021 |
| COG2996 | Uncharacterized protein conserved in bacteria | humic | 0.0124 |

| | | | |
|---|---|---|---|
| COG3001 | Fructosamine-3-kinase | clear water | 0.0338 |
| COG3008 | Paraquat-inducible protein B | humic | 0.0021 |
| COG3016 | Uncharacterized iron-regulated protein | clear water | 0.0480 |
| COG3017 | Outer membrane lipoprotein involved in outer membrane biogenesis | humic | 0.0032 |
| COG3023 | Negative regulator of beta-lactamase expression | humic | 0.0046 |
| COG3024 | Uncharacterized protein conserved in bacteria | clear water | 0.0338 |
| COG3026 | Negative regulator of sigma E activity | humic | 0.0126 |
| COG3033 | Tryptophanase | humic | 0.0245 |
| COG3038 | Cytochrome B561 | humic | 0.0046 |
| COG3049 | Penicillin V acylase and related amidases | humic | 0.0312 |
| COG3088 | Uncharacterized protein involved in biosynthesis of c-type cytochromes | humic | 0.0065 |
| COG3107 | Putative lipoprotein | humic | 0.0245 |
| COG3117 | Uncharacterized protein conserved in bacteria | humic | 0.0312 |
| COG3129 | Predicted SAM-dependent methyltransferase | humic | 0.0331 |
| COG3133 | Outer membrane lipoprotein | humic | 0.0182 |
| COG3138 | Arginine/ornithine N-succinyltransferase beta subunit | clear water | 0.0338 |
| COG3148 | Uncharacterized conserved protein | clear water | 0.0338 |
| COG3150 | Predicted esterase | humic | 0.0091 |
| COG3153 | Predicted acetyltransferase | clear water | 0.0183 |
| COG3158 | K+ transporter | humic | 0.0173 |
| COG3164 | Predicted membrane protein | humic | 0.0126 |
| COG3174 | Predicted membrane protein | humic | 0.0383 |
| COG3176 | Putative hemolysin | humic | 0.0046 |
| COG3179 | Predicted chitinase | humic | 0.0022 |
| COG3195 | Uncharacterized protein conserved in bacteria | clear water | 0.0458 |
| COG3199 | Uncharacterized conserved protein | clear water | 0.0093 |
| COG3203 | Outer membrane protein (porin) | humic | 0.0022 |
| COG3204 | Uncharacterized protein conserved in bacteria | clear water | 0.0044 |
| COG3210 | Large exoproteins involved in heme utilization or adhesion | humic | 0.0091 |
| COG3211 | Predicted phosphatase | clear water | 0.0062 |
| COG3213 | Uncharacterized protein involved in response to NO | humic | 0.0004 |
| COG3219 | Uncharacterized protein conserved in bacteria | humic | 0.0382 |
| COG3243 | Poly(3-hydroxyalkanoate) synthetase | humic | 0.0065 |
| COG3254 | Uncharacterized conserved protein | clear water | 0.0358 |
| COG3258 | Cytochrome c | humic | 0.0456 |
| COG3268 | Uncharacterized conserved protein | clear water | 0.0044 |
| COG3276 | Selenocysteine-specific translation elongation factor | clear water | 0.0044 |
| COG3284 | Transcriptional activator of acetoin/glycerol metabolism | humic | 0.0383 |
| COG3287 | Uncharacterized conserved protein | humic | 0.0061 |
| COG3292 | Predicted periplasmic ligand-binding sensor domain | humic | 0.0173 |
| COG3295 | Uncharacterized protein conserved in bacteria | humic | 0.0125 |
| COG3298 | Predicted 3'-5' exonuclease related to the exonuclease domain of PolB | humic | 0.0046 |
| COG3299 | Uncharacterized homolog of phage Mu protein gp47 | humic | 0.0017 |
| COG3305 | Predicted membrane protein | humic | 0.0076 |

| | | | |
|---|---|---|---|
| COG3306 | Glycosyltransferase involved in LPS biosynthesis | humic | 0.0173 |
| COG3315 | O-Methyltransferase involved in polyketide biosynthesis | clear water | 0.0338 |
| COG3316 | Transposase and inactivated derivatives | humic | 0.0341 |
| COG3317 | Uncharacterized lipoprotein | humic | 0.0173 |
| COG3318 | Predicted metal-binding protein related to the C-terminal domain of SecA | clear water | 0.0338 |
| COG3328 | Transposase and inactivated derivatives | humic | 0.0234 |
| COG3329 | Predicted permease | clear water | 0.0231 |
| COG3330 | Uncharacterized protein conserved in bacteria | clear water | 0.0363 |
| COG3332 | Uncharacterized conserved protein | humic | 0.0311 |
| COG3333 | Uncharacterized protein conserved in bacteria | clear water | 0.0234 |
| COG3335 | Transposase and inactivated derivatives | humic | 0.0234 |
| COG3342 | Uncharacterized conserved protein | clear water | 0.0480 |
| COG3344 | Retron-type reverse transcriptase | humic | 0.0312 |
| COG3346 | Uncharacterized conserved protein | humic | 0.0312 |
| COG3358 | Uncharacterized conserved protein | humic | 0.0456 |
| COG3369 | Uncharacterized conserved protein | clear water | 0.0338 |
| COG3378 | Predicted ATPase | humic | 0.0312 |
| COG3380 | Predicted NAD/FAD-dependent oxidoreductase | clear water | 0.0408 |
| COG3384 | Uncharacterized conserved protein | humic | 0.0046 |
| COG3385 | FOG: Transposase and inactivated derivatives | humic | 0.0312 |
| COG3391 | Uncharacterized conserved protein | humic | 0.0022 |
| COG3396 | Uncharacterized conserved protein | clear water | 0.0358 |
| COG3416 | Uncharacterized protein conserved in bacteria | clear water | 0.0338 |
| COG3425 | 3-hydroxy-3-methylglutaryl CoA synthase | clear water | 0.0183 |
| COG3426 | Butyrate kinase | humic | 0.0382 |
| COG3437 | Response regulator containing a CheY-like receiver domain and an HD-GYP domain | humic | 0.0065 |
| COG3442 | Predicted glutamine amidotransferase | humic | 0.0020 |
| COG3448 | CBS-domain-containing membrane protein | humic | 0.0091 |
| COG3453 | Uncharacterized protein conserved in bacteria | humic | 0.0312 |
| COG3463 | Predicted membrane protein | clear water | 0.0338 |
| COG3464 | Transposase and inactivated derivatives | humic | 0.0046 |
| COG3467 | Predicted flavin-nucleotide-binding protein | clear water | 0.0147 |
| COG3480 | Predicted secreted protein containing a PDZ domain | clear water | 0.0022 |
| COG3484 | Predicted proteasome-type protease | humic | 0.0413 |
| COG3491 | Isopenicillin N synthase and related dioxygenases | clear water | 0.0032 |
| COG3511 | Phospholipase C | humic | 0.0456 |
| COG3515 | Uncharacterized protein conserved in bacteria | clear water | 0.0183 |
| COG3517 | Uncharacterized protein conserved in bacteria | clear water | 0.0338 |
| COG3530 | Uncharacterized protein conserved in bacteria | humic | 0.0019 |
| COG3533 | Uncharacterized protein conserved in bacteria | humic | 0.0234 |
| COG3534 | Alpha-L-arabinofuranosidase | humic | 0.0032 |
| COG3536 | Uncharacterized protein conserved in bacteria | humic | 0.0126 |
| COG3542 | Uncharacterized conserved protein | humic | 0.0022 |
| COG3544 | Uncharacterized protein conserved in bacteria | clear water | 0.0044 |

| | | | |
|---|---|---|---|
| COG3547 | Transposase and inactivated derivatives | humic | 0.0126 |
| COG3552 | Protein containing von Willebrand factor type A (vWA) domain | clear water | 0.0022 |
| COG3554 | Uncharacterized protein conserved in bacteria | clear water | 0.0183 |
| COG3555 | Aspartyl/asparaginyl beta-hydroxylase and related dioxygenases | humic | 0.0065 |
| COG3556 | Predicted membrane protein | clear water | 0.0338 |
| COG3562 | Capsule polysaccharide export protein | clear water | 0.0183 |
| COG3565 | Predicted dioxygenase of extradiol dioxygenase family | clear water | 0.0093 |
| COG3569 | Topoisomerase IB | humic | 0.0019 |
| COG3570 | Streptomycin 6-kinase | humic | 0.0245 |
| COG3571 | Predicted hydrolase of the alpha/beta-hydrolase fold | clear water | 0.0093 |
| COG3579 | Aminopeptidase C | humic | 0.0383 |
| COG3587 | Restriction endonuclease | humic | 0.0173 |
| COG3594 | Fucose 4-O-acetylase and related acetyltransferases | clear water | 0.0044 |
| COG3599 | Cell division initiation protein | clear water | 0.0146 |
| COG3608 | Predicted deacylase | clear water | 0.0019 |
| COG3614 | Predicted periplasmic ligand-binding sensor domain | humic | 0.0124 |
| COG3616 | Predicted amino acid aldolase or racemase | clear water | 0.0126 |
| COG3621 | Patatin | clear water | 0.0338 |
| COG3623 | Putative L-xylulose-5-phosphate 3-epimerase | clear water | 0.0338 |
| COG3624 | Uncharacterized enzyme of phosphonate metabolism | humic | 0.0251 |
| COG3645 | Uncharacterized phage-encoded protein | clear water | 0.0338 |
| COG3653 | N-acyl-D-aspartate/D-glutamate deacylase | clear water | 0.0126 |
| COG3657 | Uncharacterized protein conserved in bacteria | humic | 0.0173 |
| COG3659 | Carbohydrate-selective porin | humic | 0.0016 |
| COG3664 | Beta-xylosidase | humic | 0.0383 |
| COG3666 | Transposase and inactivated derivatives | humic | 0.0233 |
| COG3668 | Plasmid stabilization system protein | humic | 0.0091 |
| COG3669 | Alpha-L-fucosidase | humic | 0.0022 |
| COG3676 | Transposase and inactivated derivatives | humic | 0.0061 |
| COG3687 | Predicted metal-dependent hydrolase | clear water | 0.0093 |
| COG3695 | Predicted methylated DNA-protein cysteine methyltransferase | clear water | 0.0338 |
| COG3696 | Putative silver efflux pump | humic | 0.0091 |
| COG3703 | Uncharacterized protein involved in cation transport | clear water | 0.0338 |
| COG3706 | Response regulator containing a CheY-like receiver domain and a GGDEF domain | humic | 0.0126 |
| COG3708 | Uncharacterized protein conserved in bacteria | humic | 0.0383 |
| COG3714 | Predicted membrane protein | clear water | 0.0183 |
| COG3724 | Succinylarginine dihydrolase | clear water | 0.0338 |
| COG3732 | Phosphotransferase system sorbitol-specific component IIBC | clear water | 0.0044 |
| COG3733 | Cu2+-containing amine oxidase | clear water | 0.0091 |
| COG3737 | Uncharacterized conserved protein | humic | 0.0233 |
| COG3741 | N-formylglutamate amidohydrolase | clear water | 0.0093 |
| COG3749 | Uncharacterized protein conserved in bacteria | humic | 0.0091 |
| COG3750 | Uncharacterized protein conserved in bacteria | humic | 0.0028 |
| COG3756 | Uncharacterized protein conserved in bacteria | humic | 0.0064 |

| | | | |
|---|---|---|---|
| COG3758 | Uncharacterized protein conserved in bacteria | clear water | 0.0093 |
| COG3760 | Uncharacterized conserved protein | clear water | 0.0338 |
| COG3772 | Phage-related lysozyme (muraminidase) | humic | 0.0413 |
| COG3773 | Cell wall hydrolyses involved in spore germination | humic | 0.0233 |
| COG3774 | Mannosyltransferase OCH1 and related enzymes | humic | 0.0126 |
| COG3777 | Uncharacterized conserved protein | humic | 0.0022 |
| COG3794 | Plastocyanin | clear water | 0.0338 |
| COG3800 | Predicted transcriptional regulator | clear water | 0.0093 |
| COG3804 | Uncharacterized conserved protein related to dihydrodipicolinate reductase | clear water | 0.0338 |
| COG3812 | Uncharacterized protein conserved in bacteria | humic | 0.0126 |
| COG3824 | Uncharacterized protein conserved in bacteria | clear water | 0.0338 |
| COG3854 | Uncharacterized protein conserved in bacteria | clear water | 0.0089 |
| COG3872 | Predicted metal-dependent enzyme | clear water | 0.0093 |
| COG3876 | Uncharacterized protein conserved in bacteria | humic | 0.0234 |
| COG3879 | Uncharacterized protein conserved in bacteria | clear water | 0.0093 |
| COG3882 | Predicted enzyme involved in methoxymalonyl-ACP biosynthesis | humic | 0.0046 |
| COG3892 | Uncharacterized protein conserved in bacteria | clear water | 0.0183 |
| COG3893 | Inactivated superfamily I helicase | humic | 0.0065 |
| COG3907 | PAP2 (acid phosphatase) superfamily protein | clear water | 0.0338 |
| COG3920 | Signal transduction histidine kinase | humic | 0.0413 |
| COG3926 | Putative secretion activating protein | humic | 0.0022 |
| COG3934 | Endo-beta-mannanase | humic | 0.0383 |
| COG3935 | Putative primosome component and related proteins | humic | 0.0251 |
| COG3941 | Mu-like prophage protein | clear water | 0.0093 |
| COG3943 | Virulence protein | humic | 0.0233 |
| COG3950 | Predicted ATP-binding protein involved in virulence | humic | 0.0245 |
| COG3963 | Phospholipid N-methyltransferase | clear water | 0.0093 |
| COG3970 | Fumarylacetoacetate (FAA) hydrolase family protein | clear water | 0.0173 |
| COG3973 | Superfamily I DNA and RNA helicases | clear water | 0.0172 |
| COG3979 | Uncharacterized protein contain chitin-binding domain type 3 | humic | 0.0126 |
| COG4091 | Predicted homoserine dehydrogenase | clear water | 0.0107 |
| COG4094 | Predicted membrane protein | clear water | 0.0183 |
| COG4095 | Uncharacterized conserved protein | humic | 0.0022 |
| COG4101 | Predicted mannose-6-phosphate isomerase | clear water | 0.0338 |
| COG4108 | Peptide chain release factor RF-3 | humic | 0.0126 |
| COG4112 | Predicted phosphoesterase (MutT family) | clear water | 0.0338 |
| COG4115 | Uncharacterized protein conserved in bacteria | humic | 0.0032 |
| COG4122 | Predicted O-methyltransferase | humic | 0.0413 |
| COG4124 | Beta-mannanase | humic | 0.0014 |
| COG4186 | Predicted phosphoesterase or phosphohydrolase | humic | 0.0091 |
| COG4188 | Predicted dienelactone hydrolase | clear water | 0.0146 |
| COG4191 | Signal transduction histidine kinase regulating C4-dicarboxylate transport system | humic | 0.0233 |
| COG4195 | Phage-related replication protein | clear water | 0.0044 |
| COG4196 | Uncharacterized protein conserved in bacteria | humic | 0.0022 |

| | | | |
|---|---|---|---|
| COG4199 | Uncharacterized protein conserved in bacteria | clear water | 0.0093 |
| COG4221 | Short-chain alcohol dehydrogenase of unknown specificity | humic | 0.0022 |
| COG4222 | Uncharacterized protein conserved in bacteria | clear water | 0.0234 |
| COG4227 | Antirestriction protein | humic | 0.0234 |
| COG4229 | Predicted enolase-phosphatase | clear water | 0.0044 |
| COG4235 | Cytochrome c biogenesis factor | humic | 0.0091 |
| COG4244 | Predicted membrane protein | clear water | 0.0338 |
| COG4259 | Uncharacterized protein conserved in bacteria | humic | 0.0254 |
| COG4261 | Predicted acyltransferase | humic | 0.0176 |
| COG4270 | Predicted membrane protein | clear water | 0.0338 |
| COG4280 | Predicted membrane protein | humic | 0.0076 |
| COG4291 | Predicted membrane protein | humic | 0.0025 |
| COG4299 | Uncharacterized conserved protein | humic | 0.0126 |
| COG4313 | Protein involved in meta-pathway of phenol degradation | humic | 0.0416 |
| COG4320 | Uncharacterized protein conserved in bacteria | humic | 0.0456 |
| COG4324 | Predicted aminopeptidase | clear water | 0.0338 |
| COG4335 | DNA alkylation repair enzyme | clear water | 0.0338 |
| COG4336 | Uncharacterized conserved protein | clear water | 0.0044 |
| COG4337 | Uncharacterized protein conserved in bacteria | clear water | 0.0183 |
| COG4370 | Uncharacterized protein conserved in bacteria | clear water | 0.0338 |
| COG4371 | Predicted membrane protein | clear water | 0.0338 |
| COG4398 | Uncharacterized protein conserved in bacteria | clear water | 0.0307 |
| COG4405 | Uncharacterized protein conserved in bacteria | clear water | 0.0183 |
| COG4412 | Uncharacterized protein conserved in bacteria | clear water | 0.0363 |
| COG4424 | Uncharacterized protein conserved in bacteria | clear water | 0.0338 |
| COG4451 | Ribulose bisphosphate carboxylase small subunit | clear water | 0.0183 |
| COG4453 | Uncharacterized protein conserved in bacteria | clear water | 0.0480 |
| COG4454 | Uncharacterized copper-binding protein | clear water | 0.0225 |
| COG4456 | Virulence-associated protein and related proteins | clear water | 0.0338 |
| COG4535 | Putative Mg2+ and Co2+ transporter CorC | humic | 0.0046 |
| COG4536 | Putative Mg2+ and Co2+ transporter CorB | humic | 0.0022 |
| COG4539 | Predicted membrane protein | clear water | 0.0093 |
| COG4564 | Signal transduction histidine kinase | humic | 0.0230 |
| COG4565 | Response regulator of citrate/malate metabolism | humic | 0.0254 |
| COG4566 | Response regulator | humic | 0.0312 |
| COG4570 | Holliday junction resolvase | humic | 0.0046 |
| COG4577 | Carbon dioxide concentrating mechanism/carboxysome shell protein | clear water | 0.0093 |
| COG4582 | Uncharacterized protein conserved in bacteria | humic | 0.0173 |
| COG4583 | Sarcosine oxidase gamma subunit | clear water | 0.0093 |
| COG4584 | Transposase and inactivated derivatives | humic | 0.0126 |
| COG4585 | Signal transduction histidine kinase | humic | 0.0046 |
| COG4628 | Uncharacterized conserved protein | humic | 0.0019 |
| COG4639 | Predicted kinase | clear water | 0.0044 |
| COG4643 | Uncharacterized protein conserved in bacteria | humic | 0.0173 |

| | | | |
|---|---|---|---|
| COG4675 | Microcystin-dependent protein | humic | 0.0312 |
| COG4683 | Uncharacterized protein conserved in bacteria | clear water | 0.0338 |
| COG4692 | Predicted neuraminidase (sialidase) | humic | 0.0173 |
| COG4717 | Uncharacterized conserved protein | humic | 0.0120 |
| COG4718 | Phage-related protein | clear water | 0.0338 |
| COG4731 | Uncharacterized protein conserved in bacteria | humic | 0.0021 |
| COG4734 | Antirestriction protein | humic | 0.0245 |
| COG4748 | Uncharacterized conserved protein | clear water | 0.0338 |
| COG4753 | Response regulator containing CheY-like receiver domain and AraC-type DNA-binding domain | humic | 0.0091 |
| COG4754 | Uncharacterized conserved protein | humic | 0.0442 |
| COG4760 | Predicted membrane protein | clear water | 0.0046 |
| COG4769 | Predicted membrane protein | humic | 0.0245 |
| COG4771 | Outer membrane receptor for ferrienterochelin and colicins | humic | 0.0022 |
| COG4772 | Outer membrane receptor for Fe3+-dicitrate | humic | 0.0065 |
| COG4775 | Outer membrane protein/protective antigen OMA87 | humic | 0.0022 |
| COG4804 | Uncharacterized conserved protein | humic | 0.0046 |
| COG4813 | Trehalose utilization protein | clear water | 0.0093 |
| COG4823 | Abortive infection bacteriophage resistance protein | humic | 0.0025 |
| COG4832 | Uncharacterized conserved protein | humic | 0.0254 |
| COG4845 | Chloramphenicol O-acetyltransferase | humic | 0.0382 |
| COG4852 | Predicted membrane protein | humic | 0.0173 |
| COG4870 | Cysteine protease | humic | 0.0126 |
| COG4875 | Uncharacterized protein conserved in bacteria with a cystatin-like fold | clear water | 0.0093 |
| COG4880 | Secreted protein containing C-terminal beta-propeller domain distantly related to WD-40 repeats | clear water | 0.0183 |
| COG4886 | Leucine-rich repeat (LRR) protein | humic | 0.0173 |
| COG4887 | Uncharacterized metal-binding protein conserved in archaea | humic | 0.0077 |
| COG4923 | Uncharacterized conserved protein | clear water | 0.0338 |
| COG4934 | Predicted protease | humic | 0.0032 |
| COG4935 | Regulatory P domain of the subtilisin-like proprotein convertases and other proteases | clear water | 0.0157 |
| COG4942 | Membrane-bound metallopeptidase | humic | 0.0234 |
| COG4943 | Predicted signal transduction protein containing sensor and EAL domains | humic | 0.0077 |
| COG4974 | Site-specific recombinase XerD | humic | 0.0022 |
| COG4982 | 3-oxoacyl-[acyl-carrier protein] reductase | clear water | 0.0183 |
| COG4983 | Uncharacterized conserved protein | humic | 0.0065 |
| COG5000 | Signal transduction histidine kinase involved in nitrogen fixation and metabolism regulation | humic | 0.0091 |
| COG5002 | Signal transduction histidine kinase | humic | 0.0065 |
| COG5009 | Membrane carboxypeptidase/penicillin-binding protein | humic | 0.0022 |
| COG5011 | Uncharacterized protein conserved in bacteria | clear water | 0.0183 |
| COG5012 | Predicted cobalamin binding protein | clear water | 0.0091 |
| COG5016 | Pyruvate/oxaloacetate carboxyltransferase | humic | 0.0022 |
| COG5108 | Mitochondrial DNA-directed RNA polymerase | clear water | 0.0183 |
| COG5280 | Phage-related minor tail protein | clear water | 0.0338 |
| COG5283 | Phage-related tail protein | clear water | 0.0215 |
| COG5285 | Protein involved in biosynthesis of mitomycin antibiotics/polyketide fumonisin | clear water | 0.0091 |

| | | | |
|---|---|---|---|
| COG5301 | Phage-related tail fibre protein | humic | 0.0022 |
| COG5302 | Post-segregation antitoxin (ccd killing mechanism protein) encoded by the F plasmid | humic | 0.0077 |
| COG5304 | Uncharacterized protein conserved in bacteria | humic | 0.0182 |
| COG5306 | Uncharacterized conserved protein | humic | 0.0312 |
| COG5310 | Homospermidine synthase | clear water | 0.0358 |
| COG5323 | Uncharacterized conserved protein | humic | 0.0091 |
| COG5328 | Uncharacterized protein conserved in bacteria | clear water | 0.0338 |
| COG5337 | Spore coat assembly protein | clear water | 0.0093 |
| COG5360 | Uncharacterized protein conserved in bacteria | humic | 0.0312 |
| COG5362 | Phage-related terminase | humic | 0.0086 |
| COG5379 | S-adenosylmethionine:diacylglycerol 3-amino-3-carboxypropyl transferase | clear water | 0.0215 |
| COG5400 | Uncharacterized protein conserved in bacteria | clear water | 0.0338 |
| COG5410 | Uncharacterized protein conserved in bacteria | humic | 0.0022 |
| COG5426 | Uncharacterized membrane protein | clear water | 0.0183 |
| COG5434 | Endopolygalacturonase | humic | 0.0022 |
| COG5438 | Predicted multitransmembrane protein | clear water | 0.0093 |
| COG5441 | Uncharacterized conserved protein | clear water | 0.0311 |
| COG5449 | Uncharacterized conserved protein | humic | 0.0014 |
| COG5450 | Transcription regulator of the Arc/MetJ class | clear water | 0.0183 |
| COG5476 | Uncharacterized conserved protein | clear water | 0.0312 |
| COG5483 | Uncharacterized conserved protein | clear water | 0.0338 |
| COG5485 | Predicted ester cyclase | clear water | 0.0093 |
| COG5488 | Integral membrane protein | humic | 0.0456 |
| COG5498 | Predicted glycosyl hydrolase | humic | 0.0126 |
| COG5517 | Small subunit of phenylpropionate dioxygenase | clear water | 0.0044 |
| COG5519 | Superfamily II helicase and inactivated derivatives | humic | 0.0173 |
| COG5527 | Protein involved in initiation of plasmid replication | humic | 0.0331 |
| COG5542 | Predicted integral membrane protein | clear water | 0.0183 |
| COG5553 | Predicted metal-dependent enzyme of the double-stranded beta helix superfamily | humic | 0.0172 |
| COG5557 | Polysulphide reductase | humic | 0.0312 |
| COG5563 | Predicted integral membrane proteins containing uncharacterized repeats | humic | 0.0032 |
| COG5580 | Activator of HSP90 ATPase | humic | 0.0382 |
| COG5587 | Uncharacterized conserved protein | humic | 0.0021 |
| COG5598 | Trimethylamine:corrinoid methyltransferase | clear water | 0.0022 |
| COG5614 | Bacteriophage head-tail adaptor | clear water | 0.0338 |
| COG5621 | Predicted secreted hydrolase | clear water | 0.0347 |
| COG5632 | N-acetylmuramoyl-L-alanine amidase | humic | 0.0022 |
| COG5652 | Predicted integral membrane protein | humic | 0.0312 |
| COG5655 | Plasmid rolling circle replication initiator protein and truncated derivatives | humic | 0.0038 |

Supplementary Table S2

| Pfam | Pathway |
|---|---|
| Photo_RC | Aerobic anoxygenic phototrophy |
| APS_kinase | Assimilatory sulfate reduction |
| ATP-sulfurylase | Assimilatory sulfate reduction |
| BChl_A | bacterial photosynthesis |
| RuBisCO_large | Calvin cycle |
| RuBisCO_small | Calvin cycle |
| COXG | CO oxidation |
| Nitr_red_alph_N | Denitrification |
| Nitrate_red_gam | Denitrification |
| NosL | Denitrification |
| Cytochrom_C552 | Dissimilatory nitrate reduction |
| APS-reductase_C | Dissimilatory sulfate reduction |
| NIR_SIR | Dissimilatory sulfate/nitrate reduction |
| DmsC | DMSO reduction |
| PFO_beta_C | Fermentation |
| RC-P840_PscD | GSB photosynthesis |
| Citrate_synt | Krebs cycle |
| MeMo_Hyd_G | Methane oxidation |
| MCR_alpha | Methanogenesis and reverse methanogenesis |
| MCR_alpha_N | Methanogenesis and reverse methanogenesis |
| MCR_beta | Methanogenesis and reverse methanogenesis |
| MCR_beta_N | Methanogenesis and reverse methanogenesis |
| MCR_gamma | Methanogenesis and reverse methanogenesis |
| AMO | Nitrification |
| AmoA | Nitrification |
| Archaeal_AmoA | Nitrification |
| AmoC | Nitrification/methane oxidation |
| Monooxygenase_B | Nitrification/methane oxidation |
| NIR_SIR_ferr | Nitrogen and sulfur assimilation |
| Gln-synt_C | Nitrogen assimilation |
| Gln-synt_N | Nitrogen assimilation |
| Glu_syn_central | Nitrogen assimilation |
| Glu_synthase | Nitrogen assimilation |
| Fer4_NifH | Nitrogen fixation |
| Nitro_FeMo-Co | Nitrogen fixation |
| Bac_GDH | Nitrogen mineralization/assimilation |
| GDH_N | Nitrogen mineralization/assimilation |
| NAD-GH | Nitrogen mineralization/assimilation |
| COX1 | oxidative phosphorylation |
| Cytochrom_C | oxidative phosphorylation |
| PRK | pentose phosphate pathway |
| COX3 | respiration |
| Cytochrome_CBB3 | respiration |
| CitF | rTCA |
| CDO_I | Sulfur mineralization |
| CdhC | Wood-Ljungdahl |
